# Neurochemical alterations of different cerebral regions in rats with myocardial ischemia-reperfusion injury based on proton nuclear magnetic spectroscopy analysis

**DOI:** 10.18632/aging.202250

**Published:** 2020-12-14

**Authors:** Mao-Hui Feng, Zhi-Xiao Li, Qian Wang, Anne Manyande, Yu-Juan Li, Shun-Yuan Li, Weiguo Xu, Hong-Bing Xiang

**Affiliations:** 1 Department of Gastrointestinal Surgery, Zhongnan Hospital, Wuhan University, Wuhan, China; 2 The Clinical Medical Research Center of Peritoneal Cancer of Wuhan, Clinical Cancer Study Center of Hubei Province, Key Laboratory of Tumor Biological Behavior of Hubei Province, Wuhan, China; 3 Departments of Anesthesiology and Pain Medicine, Tongji Hospital of Tongji Medical College, Huazhong University of Science and Technology, Wuhan, China; 4 School of Human and Social Sciences, University of West London, London, UK; 5 Department of Anesthesiology, The First Affiliated Quanzhou Hospital of Fujian Medical University, Quanzhou, China; 6 Department of Orthopedics, Tongji Hospital of Tongji Medical College, Huazhong University of Science and Technology, Wuhan, China

**Keywords:** myocardial ischemia-reperfusion injury, proton nuclear magnetic spectroscopy, thalamus, brainstem

## Abstract

Background: Recent studies have demonstrated a complex and dynamic neural crosstalk between the heart and brain. A heart-brain interaction has been described regarding cardiac ischemia, but the cerebral metabolic mechanisms involved are unknown.

Methods: Male Sprague Dawley rats were randomly allocated into 2 groups: those receiving myocardial ischemia-reperfusion surgery (IR group, n =10) and surgical controls (Con group, n=10). These patterns of metabolic abnormalities in different brain regions were assessed using proton magnetic resonance spectroscopy (PMRS).

Results: Results assessed by echocardiography showed resultant cardiac dysfunction following heart ischemia-reperfusion. Compared with the control group, the altered metabolites in the IR group were taurine and choline, and differences mainly occurred in the thalamus and brainstem.

Conclusions: Alterations in cerebral taurine and choline are important findings offering new avenues to explore neuroprotective strategies for myocardial ischemia-reperfusion injury. These results provide preliminary evidence for understanding the cerebral metabolic process underlying myocardial ischemia-reperfusion injury in rats.

## INTRODUCTION

Elucidating the connectivity and functionality of a particular heart-brain circuit is one of the most challenging research goals in cardiology and neuroscience. Various strategies have been developed to reveal brain neural networks [1–5]. Over the past decade there has been a renaissance in our understanding of heart-brain interaction; new technologies are beginning to provide key insights into metabolic crosstalk between the heart and brain in response to internal and external cues [6, 7]. Myocardial ischemia-reperfusion injury (MIRI) is known to be associated with comorbid nociceptive disorders and increased mortality risk [8–12]. The mechanisms through which MIRI is linked to these negative outcomes remains unclear, in part due to limited knowledge of the pathophysiology of MIRI itself. Some clues to the pathophysiology of myocardial ischemia may be derived from its clinical features. Impairments associated with myocardial ischemia can be categorized into nociceptive [13, 14] and affective domains [15]. The first domain is associated with brain regions that most consistently involve the amygdala, thalamus, cortex, anterior singular cortex (ACC), periaqueductal gray and rostral ventromedial medulla (RVM) [15–19]. Structures more critically involved in MIRI, may include the brainstem [19, 20] and thalamic nucleus [7, 21].

Proton magnetic resonance spectroscopy (PMRS) has helped to elucidate the heart-brain interaction at the macro level. It is an important neuroimaging modality that can quantify the concentration of specific neurochemicals in the central nervous system including the spinal cord and brain. PMRS analysis of local metabolic changes in the brain provides a quick modality for evaluating the specific neuronal activity [22], which in turn is controlled by changes in cerebral blood flow [23]. Although the underlying neuro-metabolic coupling mechanisms are still not completely understood, PMRS has already become a valued tool to detect amino acid levels and thus, brain-wide metabolic networks mapping. In the current study, we used PMRS to examine the alterations of amino acid levels in different brain regions associated with myocardial IR injury reproduced by the intermittent occlusion of the left anterior descending coronary artery [9, 24–32].

## RESULTS

### Characteristics of myocardial ischemia tissues

With regard to rats in the IR group, we observed the development of ST-segment elevation and QRS complex changes on an electrocardiogram, and the obvious cyanotic change in the myocardium of the occluded area 30 min after cardiac ischemia. Otherwise, serum cardiac troponin cTnI in the IR group was significantly increased compared to the Con group 2h after reperfusion. These results verified the performance of successful occlusion of the left anterior descending coronary artery [33].

### Cardiac function assessed by echocardiography

Average data of LVEF and LVFS were assessed by echocardiography in male SD rats. Rats subjected to MIRI exhibited significantly decreased cardiac contractile function measured by LVEF (p < 0.01) and LVFS (p < 0.001) at 2h after MIRI compared to surgical controls (Supplementary Figures 1, 2 and Supplementary Table 1), suggesting that MIRI induced cardiac dysfunction.

### PMRS-based metabolic information at different brain regions

**Figure 1:**
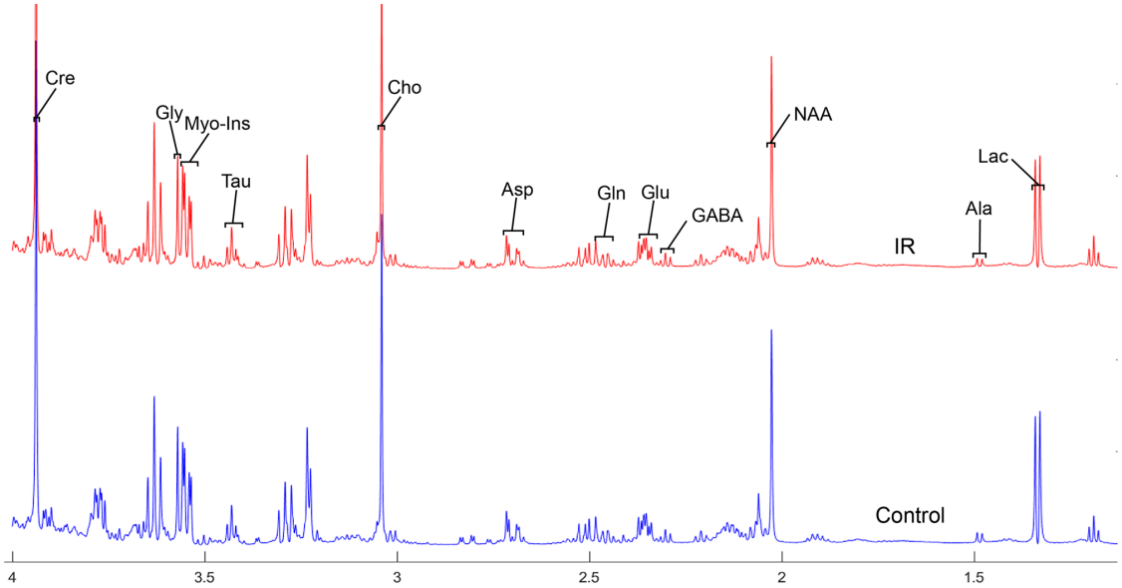
**The normalized ^1^H NMR spectra of extracts in medulla-pons after MIRI.** Note: Lac, lactate; GABA, gamma-aminobutyric acid; NAA, N-acetylaspartate; Asp, aspartate; Glu, glutamine; Gln, Glutamate; Cre, creatinine; Cho, choline; Myo, Myo-inositol; Tau, taurine; Ala, alanine; Gly, glycine.

**Figure 2:**
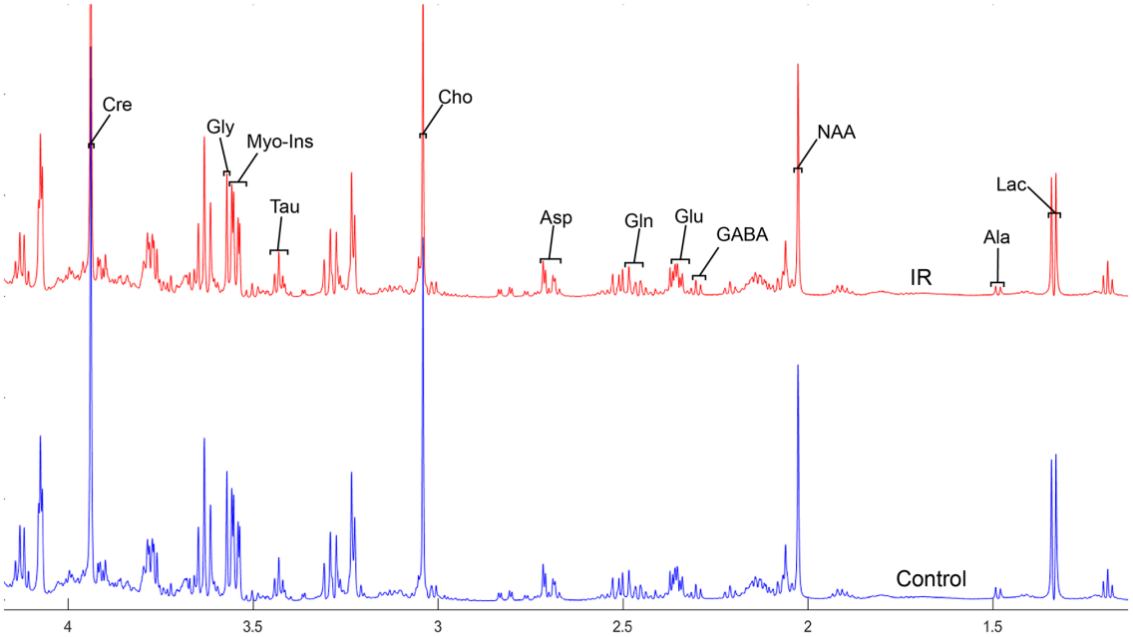
**The normalized ^1^H NMR spectra of extracts in the parietal cortex after MIRI.**

**Figure 3:**
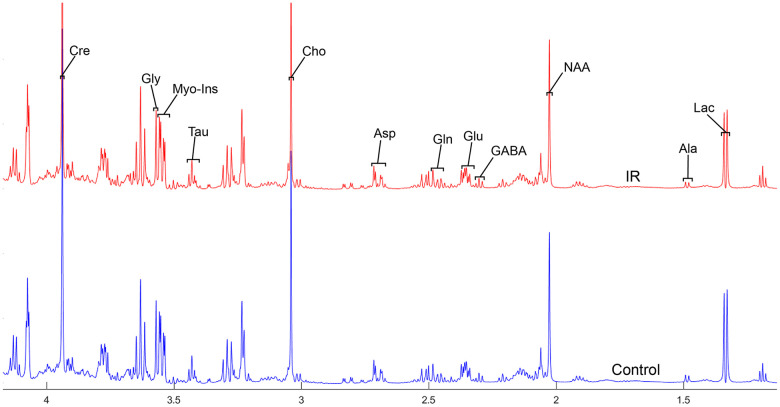
**The normalized ^1^H NMR spectra of extracts in the striatum after MIRI.**

**Figure 4:**
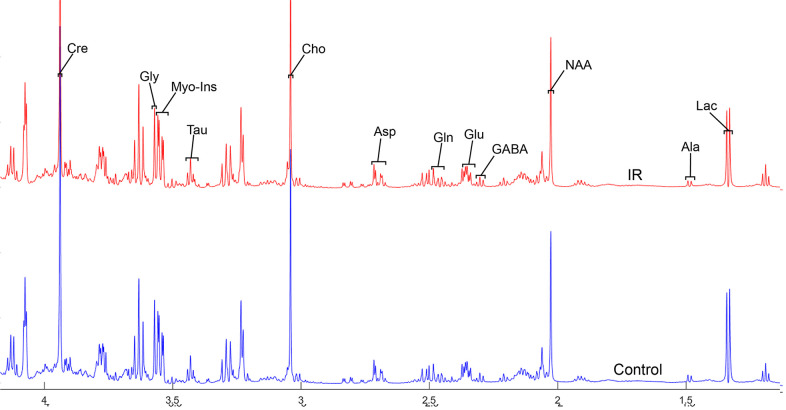
**The normalized ^1^H NMR spectra of extracts in the thalamus after MIRI.**

To address the concentrations of cerebral metabolites that may be related to myocardial IR injury, we selected four brain regions (medulla-pons, parietal cortex, striatum, and thalamus) to analyze the information on metabolites in the spectra [illustrated in Figures 1, 2, 3 and 4]. The average normalized spectra of the two groups and the basic metabolic information, including the metabolite name and the related chemical shift are shown in Figures 1, 2, 3 and 4.

### Absolute metabolite concentrations differ markedly across rat brain regions after myocardial IR injury

We dissected 12 brain regions of two groups and employed PMRS spectra to examine the level of metabolites, including Cretine, Glycine, myo_Insitol, Taurine, Choline, Asparate, Glutamine, Glutamate, GABA, NAA, Alanine, and Lactate (Table 1 and Supplementary Table 2). We found that the level of metabolites was comparable across almost all different brain regions studied, including PC, OC, TC, MID, MED-PONs, STR, HP, and THA, except for PFC, OB, CE, and HYP (Table 1 and Supplementary Table 2). Interestingly, the PC, OC, TC, STR, HP, and THA in the IR group showed a higher level of Taurine compared to the Con group, whereas the level of Taurine in the other brain regions examined (including MID, MED-PONs) was not different between the IR group and Con group.

**Table 1:** Various metabolites changes in different brain regions with MIRI in rats.

**Brain regions**	**Metabolite**	**Variety**
Medulla-Pons	**Cho**	32.1%↓ (P=0.046)
	**GABA**	16.1%↑(P=0.043)
Midbrain	**Cho**	28.6%↓ (P=0.032)
Hippocampus	**Myo-Ins**	6.3%↑(P=0.025)
	**Tau**	13.8%↑(P=0.009)
Striatum	**Tau**	14.1%↑(P=0.004)
	**Gln**	12.3%↑(P=0.011)
Thalamus	**Tau**	33.4%↑(P=0.032)
	**Cho**	40.5%↑(P=0.010)
		
Temporal cortex	**Tau**	20.7%↑(P=0.025)
	**NAA**	14.1%↑(P=0.047)
Parietal cortex	**Tau**	16.6%↑(P=0.018)
	**Cre**	8.1%↑(P=0.025)
	**Gly**	14.2%↑(P=0.016)
Occipital cortex	**Tau**	12.1%↑(P=0.026)

**Note:** The data were compared with control group. “↑”and “↓” indicates increase and decrease, respectively. Statistical analysis was performed by the Mann-Whitney test and P < 0.05 was considered significant difference. Cho, choline; GABA, gamma-aminobutyric acid; Myo-Ins, Myo- inositol; Tau, taurine; Gln, glutamine; Cre, creatinine; NAA, N-acetylaspartate; Gly, glycine.

### Changes in metabolic concentration of different cerebral regions after myocardial IR injury

**Figure 5:**
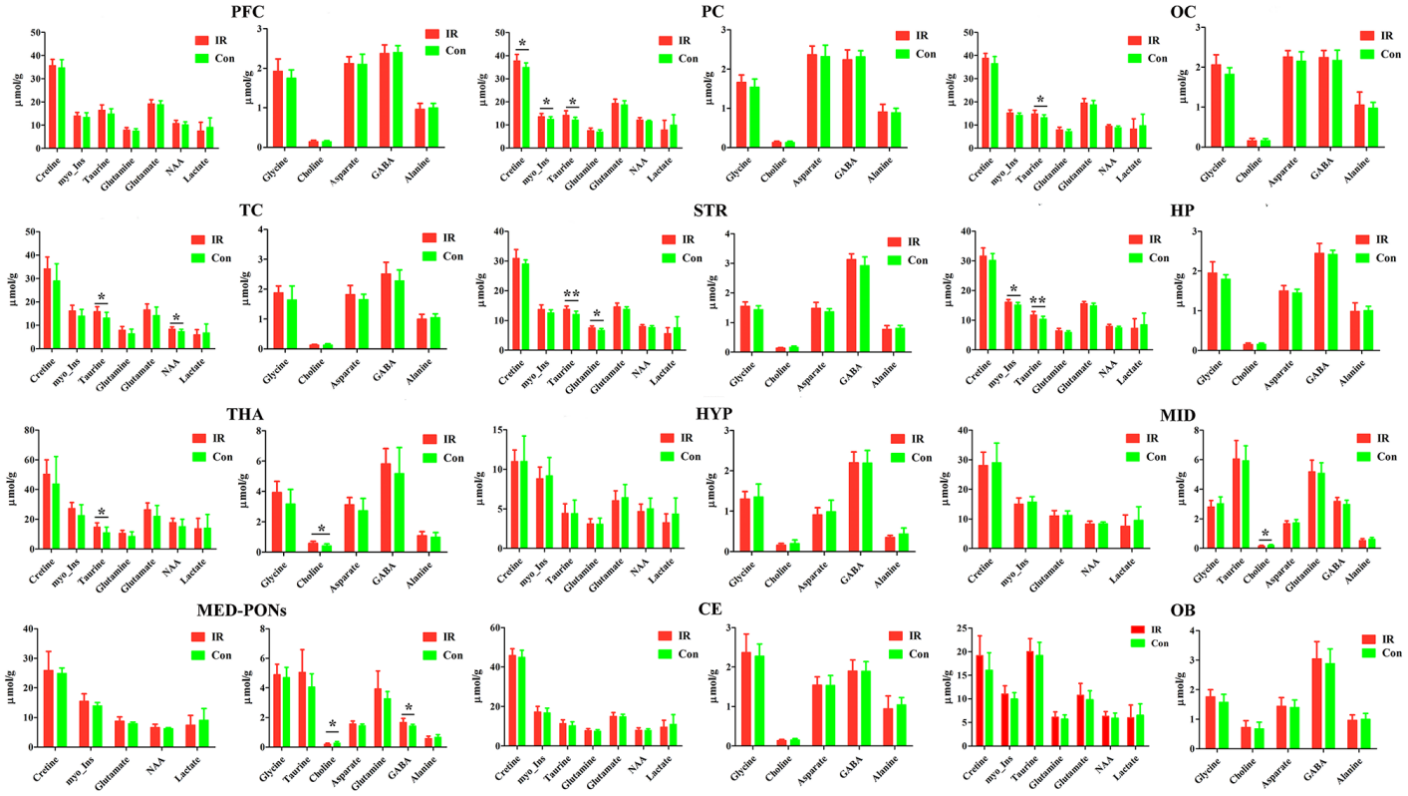
**The concentration of identified metabolites in different brain regions in control (con) and ischemia reperfusion (IR) groups using the PMRS method.** Data were presented as means ± SEM. Mann-Whitney test. *p< 0.05, **p<0.01. Note: FC, Prefrontal Cortex; PC, Parietal cortex; OC, Occipital cortex; TC, Temporal Cortex; STR, Striatum; HP, Hippocampus; HTA, Thalamus; HYP, Hypothalamus; MID, Midbrain; MED-PONs, Medulla-Pons; CE, Cerebellum; OB, Olfactory Bulbs; GABA, gamma amino acid butyric acid; NAA, N-acetyl aspartate.

In order to precisely assess cerebral changes after myocardial IR injury, absolute concentrations of all related metabolites were calculated and compared (Supplementary Table 2 and Figure 5). The average difference in normalized PMRS spectra of selected metabolites between the Con and IR groups are displayed in Figures 1, 2, 3 and 4.

Taurine is differentially expressed in various rat brain regions. In the case of Taurine, we observed higher levels in the PC (p=0.018), OC (p=0.026), TC (p=0.025), STR (p=0.004), HP (p=0.009), and THA (p=0.032) of the IR group compared to the Con group.

Otherwise, we found a higher concentration of Cretine (p=0.025) and Glycine (p=0.016) in the PC of the IR group compared to the Con group, respectively, and a higher concentration of NAA (p=0.047) in the TC of the IR group compared to Con group.

In the case of Choline, we observed lower levels in the MID (p=0.032), MED-PONs (p=0.046) of the IR group compared to the Con group, whereas higher levels in the THA (p=0.010) of the IR group compared to the Con group.

Simultaneously, we observed a higher concentration of GABA in the MED-PONs (p=0.043), glutamine in the STR (p=0.011), and myo_Insitol in the HP (p=0.025) of the IR group compared to the Con group.

### Discriminant analysis between the two groups

**Figure 6:**
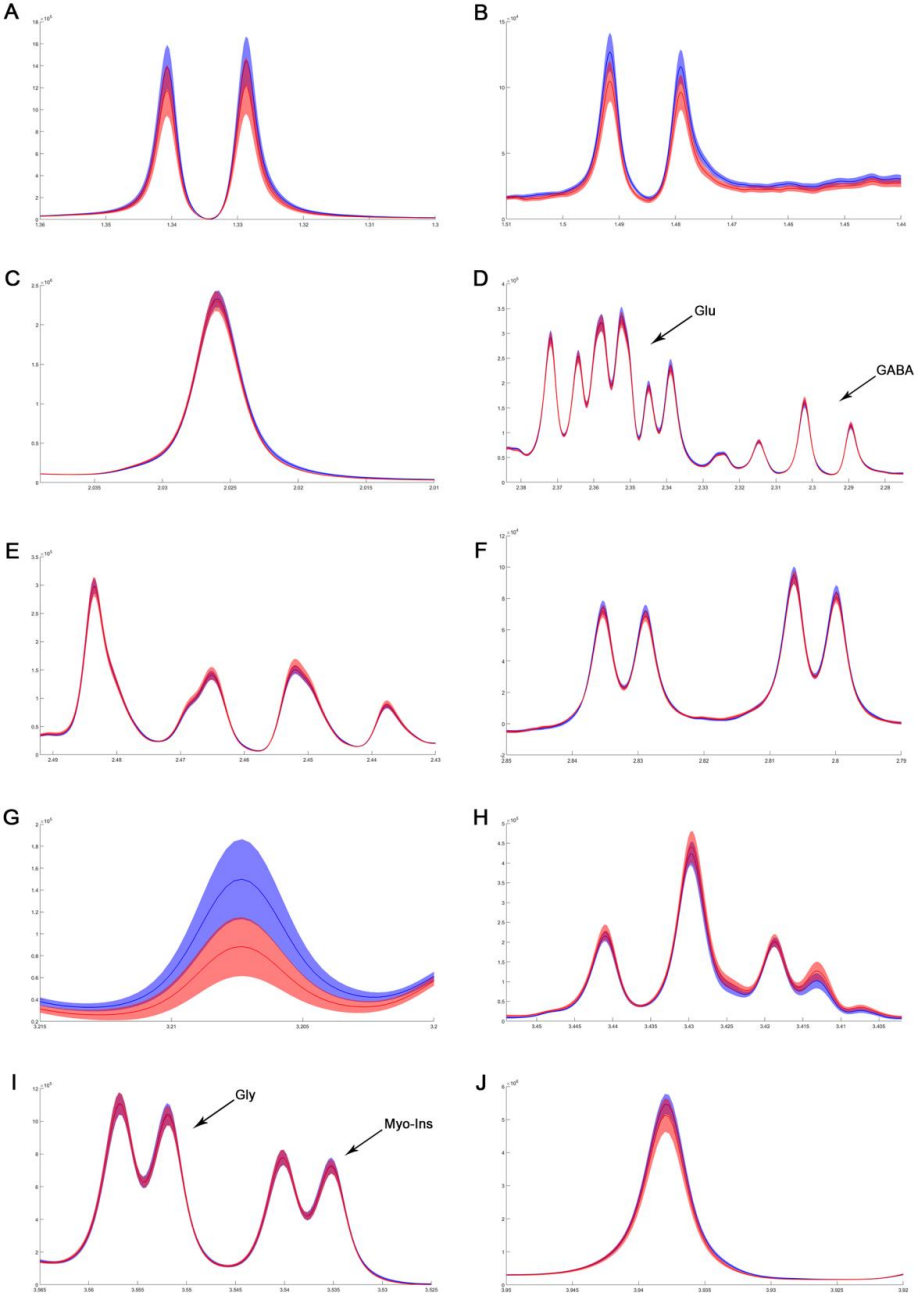
**The difference in metabolites of normalized spectra in medulla-pons after MIRI.** (**A**) Lactate; (**B**) Alanine; (**C**) N-acetylaspartate; (**D**) GABA and Glutamate; (**E**) Glutamine; (**F**) Aspartate; (**G**) Choline; (**H**) Taurine; (**I**) Myo-inositol and Glycine; (**J**) Creatinine. The spectral line and the width of its shadow represent mean and standard deviation, respectively. Control group: blue line with shadow around; IR group: red line with shadow around.

**Figure 7:**
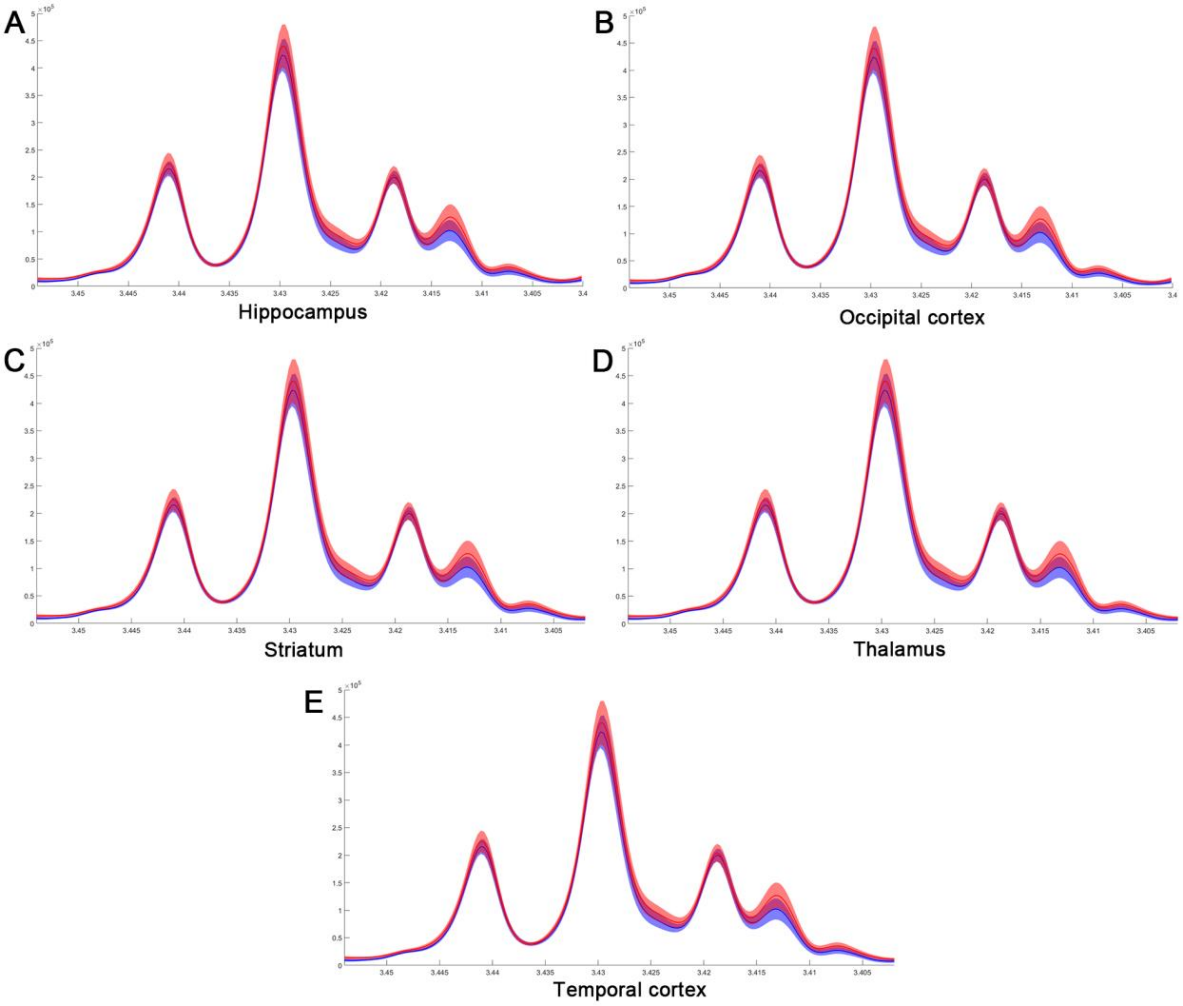
**The difference in taurine of normalized spectra in different brain regions after MIRI.** (**A**) Hippocampus; (**B**) Occipital cortex; (**C**) Striatum; (**D**) Thalamus; (**E**) Temporal cortex.

Considering the vital function of metabolites including Cretine, Glycine, myo_Insitol, Taurine, Choline, Asparate, Glutamine, Glutamate, GABA, NAA, Alanine, and Lactate in different brain regions, metabolic changes in the medulla-pons, parietal cortex, striatum, and thalamus were further investigated (Figure 6). The Taurine changes in the FC are illustrated in Figure 7. Our results demonstrate that myocardial ischemia-reperfusion injury induced increase in Taurine levels in the Hippocampus, Occipital cortex, Striatum, Thalamus, and Temporal cortex.

## DISCUSSION

Recently, significant efforts have been made to understand the alterations of amino acid levels in different brain regions and their contributions to specific metabolic disturbances of the disease [34–36]. The brain is a complex and dynamic metabolic structure for which functions are associated with the generation of energy activity in its targets [37]. Here, we aimed to understand the involvement of specific patterns of cerebral metabolites and glucose metabolism in different brain regions of animals with myocardial ischemia-reperfusion injury.

Compared to the control group, the rats in the IR group displayed a dramatic increase in the level of taurine in many brain regions including the PC, OC, TC, STR, HP, and THA, as well as a decrease in choline in the MID and MED-PONs but an increase in choline in the THA. It is well-known that taurine and creatine are neuroprotective metabolites [38–40]. Taurine is present in different brain regions and demonstrates an important inhibitory amino acid associated with extensive neuroprotective activities within the body [29, 41]. It has a functional role in osmoregulation in the brain under pathological conditions [38, 42, 43]. Jakaria et al. reported that taurine has potential therapeutic effects against different neurological disorders and protects against injuries and toxicities of the nervous system [39]. Wang et al. observed that rats with myocardial IR injury had higher concentration of taurine in the upper thoracic spinal cord, suggesting that an alteration in spinal taurine associated with IR is involved in the effects of central regulation [8]. Consistent with these findings, our result indicates that IR increased cerebral concentrations of taurine in major brain regions, suggesting a critical role of taurine in the CNS during IR proceeding.

The thalamus (THA) is one of the most consistently implicated brain region in modulating cardiac functions. Previous studies have reported that acute myocardial ischemia induces increased neuronal activity in the parafascicular nucleus of the thalamus (PFT) [14, 44]. A study by Cheng and his colleagues demonstrated that cardioprotection was induced in a mouse model of chronic neuropathic pain via the anterior nucleus of the paraventricular thalamus [21]. Guo and Yuan showed that the discharge rate of PFT was markedly increased after coronary artery ischemia [44]. While Wang and Guo documented that the nociceptive-specific neuron in the PFT had a significant increase in the discharge rate following coronary ischemia [14], suggesting that there exists an important role for the PFT in regulating the nociception associated with acute myocardial ischemia. We observed an increase in taurine and choline of the THA in the IR group compared to the Con group. These results suggest an important role of the THA in the pathogenesis and regulation of myocardial IR injury.

In summary, we explored metabolic alterations of different cerebral regions in rats with myocardial ischemia-reperfusion injury by means of the PMRS. The metabolites that fluctuated most in the various cerebral regions were two critical amino-acids, taurine and choline. Our results provide preliminary evidence for understanding the cerebral metabolic process underlying myocardial ischemia-reperfusion injury in rats.

## Material and Methods

### Animal care

Animals were cared for according to the protection of vertebrate animals used for experimental purposes and institutional guidelines 86/609/CEE, November 24, 1986. The experiments were approved by the animal care committee of Tongji Medical College, Huazhong University of Science and Technology (No.TJ20150804) and were performed according to the ARRIVE (Animal Research: Reporting In Vivo Experiments) guidelines. Male Sprague Dawley (SD) rats weighing 250-300 g at the beginning of the experiment were housed at 22±1° C under a 12-hr light-dark cycle. They had free access to water and food until surgery. All procedures were conducted in an isolated quiet room to reduce variance.

### Experimental design

Rats were randomly assigned to one of the two groups: those receiving myocardial ischemia-reperfusion surgery (IR group, n =10) and surgical controls (Con group, n=10). 2h after ischemia-reperfusion, all animals were prepared for the PMRS study.

### Surgical procedure and myocardial IR injury model in rats

Myocardial IR surgeries were performed as previously described [9, 24, 25]. Briefly, prior to surgery, each rat was deeply anaesthetized and given atropine (0.25-0.3 ml of a 50 mg/ml solution intraperitoneally) to reduce salivation. After tracheal intubation, an invasive incision was made to expose the heart at the fourth intercostal space. The left anterior descending (LAD) coronary artery was then located and ligatured until myocardial ischemia occurred which was indicated by visualizing a marked epicardial cyanosis. After 30 min of myocardial ischemia, the trap of the left anterior artery was opened. Reperfusion was allowed for 2 h. The surgical control group received the same surgical procedure, without any occlusion of the coronary artery and reperfusion. Rats in IR groups were monitored to confirm ST segment elevation during myocardial ischemia by the electrocardiogram. Cardiac Troponin I (cTnl) in two groups was measured 2h after reperfusion.

### Echocardiography measurements

Cardiac function was assessed after 30 min of LAD occlusion and 2 h of reperfusion using transthoracic echocardiography measurements [26]. In brief, maintaining a steady-state sedation level with 1.5%–2.1% (v/v) isoflurane and 0.5 L/min 100% O_2_ throughout the procedure, the anesthetized rats were placed in a supine position on top of a heating pad. After the pain reflex disappeared, a two-dimensional M-mode echocardiogram was used to obtain stable images via the parasternal long axis view for measuring left ventricular (LV) end-systolic diameter and LV end-diastolic diameter according to previously described data [27]. LV ejection fraction (EF) and LV fractional shortening (FS) were calculated. All data were analyzed by an observer who was blinded to the two groups.

### Brain sample preparation for the PMRS study

**Figure 8:**
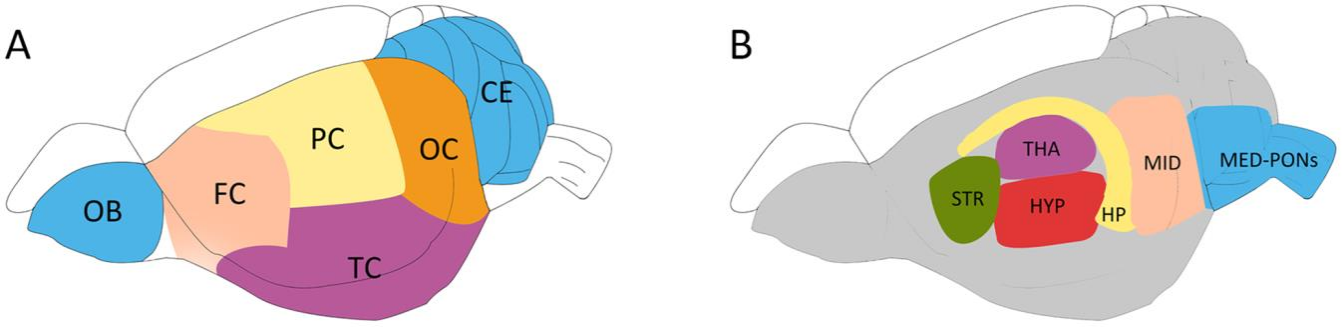
**Schematic diagram showing the rat brain regions examined using proton NMR.** Blue codes show the olfactory bulb (OB), cerebellum (CE) and cerebral cortical regions (**A**) and subcortical areas (**B**) studied. The OB and the cerebellum (CE) were first sampled, followed by the hippocampus (HP), the thalamus (THA) and the striatum (STR). The hypothalamus (HYP), the Midbrain (MID), and Medulla-Pons (MED-PONs) were discarded. The cerebral cortex tissues were coronally cut into four identical parts along the axial axis to represent the frontal cortex (FC), parietal cortex (PC), Temporal cortex (TC), and occipital cortex (OC).

To minimize the impact of post-mortem changes on cerebral metabolites [28], the anesthetized rat was microwaved using our previous method [29]. After euthanasia, the rat brain was quickly removed according to the Allen Brain Atlas [30] and dissected into 12 different regions (as indicated in Figure 8) [31]. Briefly, the olfactory bulb (OB) and the cerebellum (CE) were first sampled, followed by the hippocampus (HP), the thalamus (THA) and the striatum (STR). The hypothalamus (HYP), midbrain (MID) and medulla-pons (MED-PONs) were removed. After the entorhinal cortex was dissected, the remaining cerebral cortex tissue was coronally cut into four identical parts along the axial axis to represent the frontal cortex (FC), parietal cortex (PC), temporal cortex (TC), and occipital cortex (OC). These regional cerebral tissues were immediately weighed and stored at -80° C for further processing.

### PMRS treatment and data processing

Protocols for cerebral tissue extraction, PMRS spectrum acquirement and PMRS data processing were the same as those described in our previous study [29, 30, 32]. Detailed methods are displayed in the Supplementary Materials. The 12 related peak areas were calculated separately (Figure 8).

### Statistical analysis

Statistical data are shown as the mean ± standard error of the mean (SEM). Analysis was performed using GraphPad Prism 6.0. Comparisons between the two groups were performed using the Mann-Whitney U test. The P values of less than 0.05 were considered statistically significant.

## Supplementary Material

Supplementary Materials

Supplementary Figures

Supplementary Table 1

Supplementary Table 2
