# Supplementary material for: Neurochemical alterations of different cerebral regions in rats with myocardial ischemia-reperfusion injury based on proton nuclear magnetic spectroscopy analysis
Source: Aging (Albany NY). 2020 Dec 14;13(2):2294–309. doi: 10.18632/aging.202250 (PMC7880342; doi:10.18632/aging.202250)
Supplement: Supplementary Materials [file aging-13-202250-s001.pdf]

SUPPLEMENTARY MATERIALS

Supplementary Information

The protocol for tissue extraction was the same as that in our previous study [1]. Briefly, HCl/methanol (0.1 mol/L, 100 µL) was added to the frozen tissue and homogenized for 1.5 min at 20 Hz (Tissuelyser II, Qiagen, Germany). Ice-cold 60% ethanol (800 µL) was further added and the mixture homogenized again, before centrifugation at 14,000 g for 10 min. The supernatant was then collected. The extraction steps were repeated twice with 800 µL 60% ethanol to extract the metabolites remaining in the sediment. All the supernatants were collected and desiccated in a centrifugal drying apparatus (Thermo Scientific 2010, Germany) and freezing vacuum dryer (Thermo Scientific).

The dried product was preserved for further NMR studies. The dried product was successively dissolved in 60 µL D_2O (containing the inner standard, 3-(trimethylsilyl) propionic-2, 2, 3, 3-d_4 acid sodium salt (TSP, 120 mg/ L; 269913-1G, Sigma-Aldrich)) and 540 µL phosphate buffer (pH 7.2). The solution was mixed in a high-speed vortex and centrifuged at 14,000 g for 15 min, and the supernatant was withdrawn and transferred to an NMR tube.

Acquisition of PMRS

PMRS spectra were acquired as in previous studies [1, 2]. The extracted samples were measured with a Bruker Avance III 600 MHz NMR spectrometer (298 K) equipped with an inverse cryogenic probe (Bruker BioSpin, Germany). The spectra were acquired with a standard Watergate pulse sequence [3]. The following acquisition parameters were set for every sample: p1 (90 °C pulse), 8.35 µs; number of scans, 256; spectral width, 20 ppm; dummy scans, 8; number of free-induction decay points, 32 K.

PMRS data processing

All PMRS were processed and analyzed with TopSpin (Version 2.1, Bruker BioSpin) and a home-made software NMRSpec [4]. First, the phase correction and baseline distortion were manually completed in TopSpin. Then the corrected spectra were imported into NMRSpec for spectrum alignment, peak extraction, spectral integration, and the integration of chemical-related peaks. This software has been used in several metabolomics studies [1, 5, 6]. The chemical shifts of major amino-acids were distributed in the range of 1.20–4.46 ppm, so this gap was extracted for further analysis. First of all, the areas of all peaks (area under the curve) in this gap were automatically calculated for further statistical analysis [1]. To compensate for the different concentrations, each peak area was normalized to the sum of all the peak areas in this gap of its own spectrum prior to the discriminant analysis [2, 7, 8]. Furthermore, the absolute concentrations (lmol/g wet weight) of the identified metabolites were calculated with the related peak areas in spectra from the samples, information on the internal standards (TSP, such as concentration and proton number), and specimen weight. The calculation was as follows:

$$C_{met} = \frac{A_{met} / (R_{met} * N_H)}{A_{TMSP}} * (C_{TMSP} * V_{TMSP}) * 9/Wt$$

where Amet and ATSP are the relative areas of the peaks of the detected metabolites and TSP, and R_{met} is a constant for a specific metabolite calculated as the ratio between the partial NMR signal of the standard metabolite in selected regions (almost pure chemical signal) in a real sample and the whole proton signal in the standard spectrum; NH is the number of protons of the metabolite within the area A_{met}; C_{TSP} and V_{TSP} are the concentration and volume of TSP standard solution added to the NMR tube; *Wt* is the total weight of the wet specimen and 9 is the number of protons in the TSP.

Supplementary References

1. Liu T, He Z, Tian X, Kamal GM, Li Z, Liu Z, Liu H, Xu F, Wang J, Xiang H. Specific patterns of spinal metabolites underlying α-Me-5-HT-evoked pruritus compared with histamine and capsaicin assessed by proton nuclear magnetic resonance spectroscopy. Biochim Biophys Acta Mol Basis Dis. 2017; 1863:1222–30. https://doi.org/10.1016/j.bbadis.2017.03.011 PMID:28344131

2. Du H, Fu J, Wang S, Liu H, Zeng Y, Yang J, Xiong S. 1H-NMR metabolomics analysis of nutritional components from two kinds of freshwater fish brain extracts. RSC Advances. 2018; 8:19470–78. https://doi.org/10.1039/C8RA02311E

3. Liu ML, Mao XA, Ye CH, Huang H, Nicholson JK, Lindon JC. Improved WATERGATE pulse sequences for solvent suppression in NMR spectroscopy. J Magn Reson. 1998; 132:125–29. https://doi.org/10.1006/jmre.1998.1405

4. Liu Y, Cheng J, Liu HL, Deng YH, Wang J, Xu FQ. NMRSpec: an integrated software package for processing and analyzing one dimensional nuclear magnetic resonance spectra. Chemom Intell Lab Syst. 2017; 162:142–48. https://doi.org/10.1016/j.chemolab.2017.01.005

5. Kamal GM, Wang X, Bin Yuan, Wang J, Sun P, Zhang X, Liu M. Compositional differences among Chinese soy sauce types studied by (13)C NMR spectroscopy coupled with multivariate statistical analysis. Talanta. 2016; 158:89–99. https://doi.org/10.1016/j.talanta.2016.05.033 PMID:27343582

6. Kamal GM, Yuan B, Hussain AI, Wang J, Jiang B, Zhang X, Liu M. (13)C-NMR-Based Metabolomic Profiling of Typical Asian Soy Sauces. Molecules. 2016; 21:1168. https://doi.org/10.3390/molecules21091168 PMID:27598115

7. Wang J, Zeng HL, Du H, Liu Z, Cheng J, Liu T, Hu T, Kamal GM, Li X, Liu H, Xu F. Evaluation of metabolites extraction strategies for identifying different brain regions and their relationship with alcohol preference and gender difference using NMR metabolomics. Talanta. 2018; 179:369–76. https://doi.org/10.1016/j.talanta.2017.11.045 PMID:29310246

8. Zhang L, Wang L, Hu Y, Liu Z, Tian Y, Wu X, Zhao Y, Tang H, Chen C, Wang Y. Selective metabolic effects of gold nanorods on normal and cancer cells and their application in anticancer drug screening. Biomaterials. 2013; 34:7117–26. https://doi.org/10.1016/j.biomaterials.2013.05.043 PMID:23787109
