# Supplementary material for: Neurochemical alterations of different cerebral regions in rats with myocardial ischemia-reperfusion injury based on proton nuclear magnetic spectroscopy analysis
Source: Aging (Albany NY). 2020 Dec 14;13(2):2294–309. doi: 10.18632/aging.202250 (PMC7880342; doi:10.18632/aging.202250)
Supplement: Supplementary Figures [file aging-13-202250-s002.pdf]

SUPPLEMENTARY FIGURES

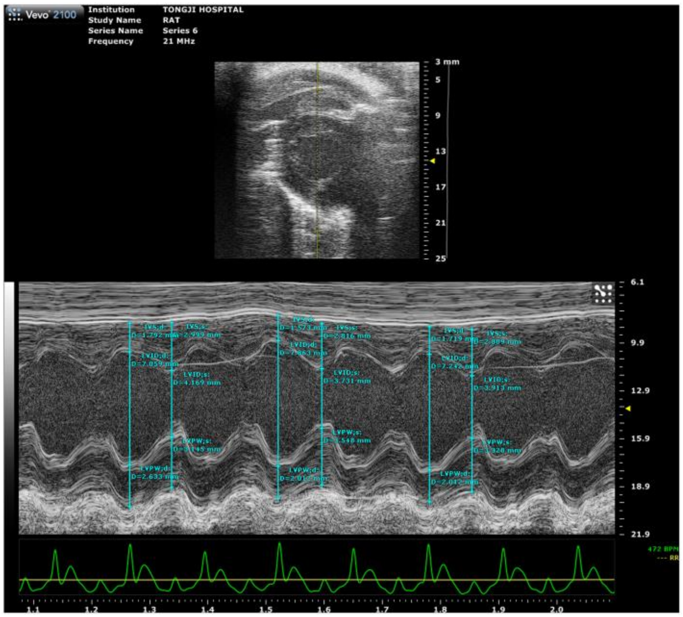

Supplementary Figure 1. Cardiac function assessed by M-mode echocardiograms in the Control group. For echocardiographic analyses, images were recorded in parasternal long-axis projections with guided one-dimensional M-mode recordings at the mid ventricular level. Standard measurements of inter-ventricular septum (IVS), left ventricular internal diameter (LVID) and left ventricular posterior wall (LVPW) were performed in systole and diastole in parasternal long-axis projection.

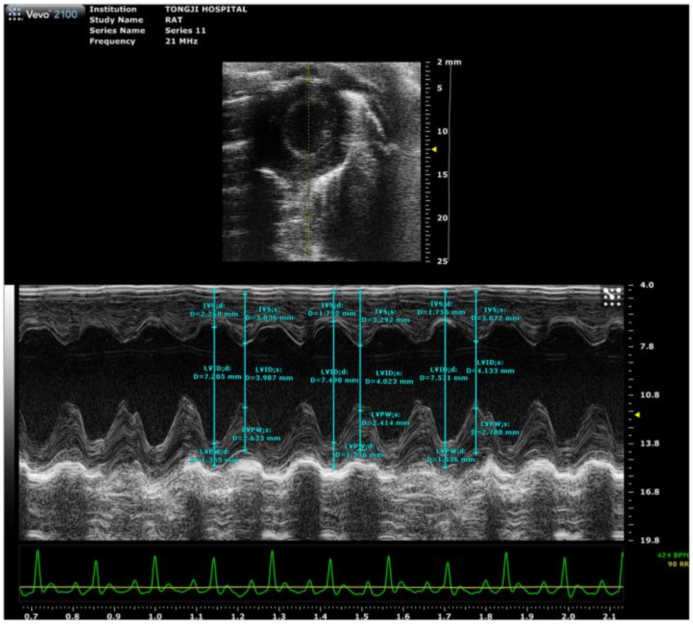

Supplementary Figure 2. Cardiac function assessed by M-mode echocardiograms in the IR group. For echocardiographic analyses, images were recorded in parasternal long-axis projections with guided one-dimensional M-mode recordings at the mid ventricular level. Standard measurements of inter-ventricular septum (IVS), left ventricular internal diameter (LVID) and left ventricular posterior wall (LVPW) were performed in systole and diastole in parasternal long-axis projection.
