# Supplementary material for: Neurochemical alterations of different cerebral regions in rats with myocardial ischemia-reperfusion injury based on proton nuclear magnetic spectroscopy analysis
Source: Aging (Albany NY). 2020 Dec 14;13(2):2294–309. doi: 10.18632/aging.202250 (PMC7880342; doi:10.18632/aging.202250)
Supplement: Supplementary Table 1 [file aging-13-202250-s003.pdf]

Supplementary Table 1. LVEF(%) and LVFS(%) of two groups 2h after MIRI.

	Baseline	MIRI 2h
LVEF(%)		
Con Group	72.42 ± 3.193	69.94 ± 2.478
IR Group	75.49 ± 1.512	65.65 ± 2.478*
LVFS(%)		
Con Group	42.15 ± 2.878	38.81 ± 2.849
IR Group	43.31 ± 1.077	34.16 ± 1.353**

Averaged data ejection fraction (LVEF) and fractional shortening (LVFS) assessed by echocardiography in male SD rats subjected to control or IR injury (30 min ischemia and 2h reperfusion). n = 4 rats per group. Results were expressed as mean ± SEM, *P < 0.01, **P < 0.001. Student's t-test as compared to Con group basal state.
