# Supplementary material for: Neurochemical alterations of different cerebral regions in rats with myocardial ischemia-reperfusion injury based on proton nuclear magnetic spectroscopy analysis
Source: Aging (Albany NY). 2020 Dec 14;13(2):2294–309. doi: 10.18632/aging.202250 (PMC7880342; doi:10.18632/aging.202250)
Supplement: Supplementary Table 2 [file aging-13-202250-s004.docx]

**Supplementary Table 2. The level of metabolites in 12 brain regions of two groups.**

1. Olfactory Bulbs (OB)

|  | MIR(μmol/g) | Control(μmol/g) | *p* value |
| --- | --- | --- | --- |
| Cretine | 19.15 ±3.99 | 16.03±3.57 | 0.107 |
| Glycine | 1.75 ±0.23 | 1.58±0.25 | 0.150 |
| myo_Insitol | 11.00±1.65 | 9.94±1.30 | 0.156 |
| Taurine | 20.03±2.60 | 19.16±2.68 | 0.506 |
| Choline | 0.72 ±0.22 | 0.68±0.21 | 0.671 |
| Asparate | 1.44±0.28 | 1.40±0.24 | 0.746 |
| Glutamine | 6.11±1.04 | 5.68±0.87 | 0.368 |
| Glutamate | 10.77±2.34 | 9.79±1.83 | 0.346 |
| GABA | 3.04 ±0.55 | 2.89±0.46 | 0.538 |
| NAA | 6.31±0.94 | 5.87±1.07 | 0.383 |
| Alanine | 0.96 ±0.17 | 1.00±0.19 | 0.674 |
| Lactate | 6.01±2.53 | 6.50±2.31 | 0.680 |

Data are shown as Mean±SD.

1. Prefrontal Cortex (PFC)

|  | MIR(μmol/g) | Control(μmol/g) | *p* value |
| --- | --- | --- | --- |
| Cretine | 35.71±2.50 | 34.74±3.32 | 0.509 |
| Glycine | 1.93±0.29 | 1.75±0.20 | 0.159 |
| myo_Insitol | 13.96±1.47 | 13.38±1.89 | 0.495 |
| Taurine | 16.47±2.17 | 14.90±2.09 | 0.146 |
| Choline | 0.14±0.03 | 0.14±0.02 | 0.965 |
| Asparate | 2.12±0.16 | 2.10±0.24 | 0.834 |
| Glutamine | 7.88±1.00 | 7.51±0.91 | 0.436 |
| Glutamate | 19.20±1.70 | 18.90±1.57 | 0.713 |
| GABA | 2.37±0.21 | 2.40±0.16 | 0.779 |
| NAA | 10.76±1.27 | 10.19±1.19 | 0.356 |
| Alanine | 0.96±0.14 | 1.00±0.11 | 0.528 |
| Lactate | 7.48±3.52 | 9.12±3.81 | 0.371 |

Data are shown as Mean±SD.

3. Parietal Cortex

|  | MIR(μmol/g) | Control(μmol/g) | *p* value |
| --- | --- | --- | --- |
| Cretine | 37.76 ±2.65 | 34.94 ±1.85 | **0.025** |
| Glycine | 1.69 ±0.17 | 1.48 ±0.14 | **0.016** |
| myo_Insitol | 13.62±1.22 | 12.45 ±1.02 | 0.053 |
| Taurine | 14.15 ±1.87 | 12.14 ±1.09 | **0.018** |
| Choline | 0.13 ±0.02 | 0.14 ±0.03 | 0.472 |
| Asparate | 2.40 ±0.19 | 2.25 ±0.25 | 0.185 |
| Glutamine | 7.57±0.98 | 6.94 ±0.86 | 0.195 |
| Glutamate | 19.31±1.76 | 18.69 ±1.68 | 0.478 |
| GABA | 2.25±0.25 | 2.27 ±0.14 | 0.810 |
| NAA | 12.12±0.97 | 11.53 ±0.44 | 0.139 |
| Alanine | 0.89±0.19 | 0.90±0.11 | 0.916 |
| Lactate | 7.79±3.97 | 9.93±4.24 | 0.934 |

Data are shown as Mean±SD.

4. Occipital Cortex (OC)

|  | MIR(μmol/g) | Control(μmol/g) | *p* value |
| --- | --- | --- | --- |
| Cretine | 38.83±2.00 | 36.57 ±2.84 | 0.078 |
| Glycine | 2.03 ±0.23 | 1.88 ±0.21 | 0.171 |
| myo_Insitol | 15.28 ±1.22 | 14.27 ±0.86 | 0.064 |
| Taurine | 14.82 ±1.54 | 13.22 ±1.16 | 0.026 |
| Choline | 0.15 ±0.05 | 0.17 ±0.06 | 0.320 |
| Asparate | 2.27 ±0.15 | 2.15 ±0.21 | 0.199 |
| Glutamine | 7.96 ±1.09 | 7.29 ±0.82 | 0.171 |
| Glutamate | 19.52 ±1.79 | 18.76 ±1.75 | 0.391 |
| GABA | 2.25 ±0.18 | 2.17 ±0.22 | 0.431 |
| NAA | 9.51 ±0.64 | 8.88 ±0.67 | 0.065 |
| Alanine | 1.03 ±0.32 | 1.00 ±0.16 | 0.834 |
| Lactate | 8.35 ±4.12 | 9.81 ±4.64 | 0.505 |

Data are shown as Mean±SD.

5. Temporal Cortex (TC)

|  | MIR(μmol/g) | Control(μmol/g) | *p* value |
| --- | --- | --- | --- |
| Cretine | 34.17 ±4.69 | 29.03 ±6.86 | 0.115 |
| Glycine | 1.88 ±0.21 | 1.64 ±0.44 | 0.209 |
| myo_Insitol | 16.14 ±2.22 | 13.92 ±2.74 | 0.108 |
| Taurine | 15.86 ±1.95 | 13.14 ±2.24 | **0.025** |
| Choline | 0.13 ±0.02 | 0.13 ±0.04 | 0.866 |
| Asparate | 1.82 ±0.29 | 1.65 ±0.17 | 0.187 |
| Glutamine | 7.90 ±1.47 | 6.38 ±1.86 | 0.101 |
| Glutamate | 16.59 ±2.36 | 14.24 ±3.31 | 0.139 |
| GABA | 2.51 ±0.37 | 2.28 ±0.34 | 0.231 |
| NAA | 8.33 ±0.87 | 7.30 ±0.96 | **0.047** |
| Alanine | 0.99 ±0.16 | 1.05 ±0.12 | 0.424 |
| Lactate | 5.97 ±2.07 | 6.75 ±3.60 | 0.617 |

Data are shown as Mean±SD.

6. Striatum

|  | MIR(μmol/g) | Control(μmol/g) | *p* value |
| --- | --- | --- | --- |
| Cretine | 30.90 ±2.79 | 29.04 ±1.27 | 0.095 |
| Glycine | 1.55 ±0.13 | 1.44 ±0.12 | 0.103 |
| myo_Insitol | 13.72 ±1.39 | 12.59 ±0.94 | 0.072 |
| Taurine | 13.77 ±0.98 | 12.07 ±1.03 | **0.004** |
| Choline | 0.14 ±0.02 | 0.16 ±0.04 | 0.110 |
| Asparate | 1.49 ±0.18 | 1.37 ±0.09 | 0.123 |
| Glutamine | 7.51 ±0.58 | 6.69 ±0.56 | **0.011** |
| Glutamate | 14.58 ±1.18 | 13.78 ±0.76 | 0.123 |
| GABA | 3.13 ±0.18 | 2.92 ±0.28 | 0.102 |
| NAA | 7.98 ±0.60 | 7.60 ±0.63 | 0.241 |
| Alanine | 0.77 ±0.12 | 0.81 ±0.09 | 0.484 |
| Lactate | 5.58 ±1.88 | 7.57 ±3.48 | 0.188 |

Data are shown as Mean±SD.

7. Hippocampus

|  | MIR(μmol/g) | Control(μmol/g) | *p* value |
| --- | --- | --- | --- |
| Cretine | 31.63±2.61 | 30.17±2.17 | 0.227 |
| Glycine | 1.95±0.26 | 1.80±0.10 | 0.121 |
| myo_Insitol | 16.11±0.78 | 15.15±0.83 | **0.025** |
| Taurine | 11.78±1.08 | 10.35±0.92 | **0.009** |
| Choline | 0.15±0.03 | 0.15±0.03 | 0.809 |
| Asparate | 1.50±0.13 | 1.45±0.08 | 0.342 |
| Glutamine | 6.41±0.77 | 5.93±0.45 | 0.129 |
| Glutamate | 15.57±0.70 | 14.89±0.82 | 0.085 |
| GABA | 2.45±0.23 | 2.42±0.10 | 0.761 |
| NAA | 7.92±0.63 | 7.46±0.42 | 0.088 |
| Alanine | 0.98±0.20 | 1.00±0.10 | 0.799 |
| Lactate | 7.29±3.00 | 8.52±3.62 | 0.459 |

Data are shown as Mean±SD.

8. Thalamus

|  | MIR(μmol/g) | Control(μmol/g) | *p* value |
| --- | --- | --- | --- |
| Cretine | 50.34±9.07 | 43.76±17.47 | 0.351 |
| Glycine | 3.93±0.69 | 3.18±0.90 | 0.071 |
| myo_Insitol | 27.27±3.80 | 22.51±6.87 | 0.100 |
| Taurine | 14.65±2.81 | 10.98±3.56 | **0.032** |
| Choline | 0.59±0.12 | 0.42±0.12 | **0.010** |
| Asparate | 3.13±0.44 | 2.73±0.77 | 0.216 |
| Glutamine | 10.50±1.94 | 8.54±2.79 | 0.114 |
| Glutamate | 26.53±4.25 | 22.03±6.88 | 0.128 |
| GABA | 5.82±0.95 | 5.17±1.64 | 0.341 |
| NAA | 17.76±2.66 | 15.11±4.67 | 0.175 |
| Alanine | 1.09±0.25 | 0.99±0.29 | 0.446 |
| Lactate | 13.62±6.63 | 14.07±8.71 | 0.909 |

Data are shown as Mean±SD.

9. Hypothalamus

|  | MIR(μmol/g) | Control(μmol/g) | *p* value |
| --- | --- | --- | --- |
| Cretine | 10.98±1.36 | 10.99±3.05 | 0.995 |
| Glycine | 1.30±0.17 | 1.35±0.30 | 0.686 |
| myo_Insitol | 8.82±1.38 | 9.19±2.19 | 0.701 |
| Taurine | 4.44±1.11 | 4.42±1.61 | 0.979 |
| Choline | 0.16±0.04 | 0.20±0.09 | 0.265 |
| Asparate | 0.91±0.16 | 0.99±0.27 | 0.514 |
| Glutamine | 3.11±0.59 | 3.07±0.72 | 0.928 |
| Glutamate | 6.05±1.16 | 6.44±1.56 | 0.588 |
| GABA | 2.20±0.25 | 2.19±0.30 | 0.954 |
| NAA | 4.67±0.88 | 5.02±1.26 | 0.540 |
| Alanine | 0.35±0.04 | 0.44±0.14 | 0.152 |
| Lactate | 3.27±1.03 | 4.35±1.91 | 0.190 |

Data are shown as Mean±SD.

10. Midbrain

|  | MIR(μmol/g) | Control(μmol/g) | *p* value |
| --- | --- | --- | --- |
| Cretine | 28.08±4.23 | 29.01±6.27 | 0.728 |
| Glycine | 2.81±0.41 | 3.03±0.43 | 0.299 |
| myo_Insitol | 15.03±1.92 | 15.75±1.71 | 0.430 |
| Taurine | 6.05±1.17 | 5.94±0.96 | 0.831 |
| Choline | 0.15±0.05 | 0.21±0.04 | **0.032** |
| Asparate | 1.66±0.18 | 1.72±0.21 | 0.532 |
| Glutamine | 5.18±0.74 | 5.09±0.67 | 0.784 |
| Glutamate | 11.06±1.64 | 11.26±1.40 | 0.790 |
| GABA | 3.18±0.24 | 2.98±0.27 | 0.123 |
| NAA | 8.30±0.88 | 8.37±0.54 | 0.842 |
| Alanine | 0.52±0.12 | 0.62±0.11 | 0.099 |
| Lactate | 7.56±3.58 | 9.54±4.33 | 0.323 |

Data are shown as Mean±SD.

11. Medulla-Pons

|  | MIR(μmol/g) | Control(μmol/g) | *p* value |
| --- | --- | --- | --- |
| Cretine | 25.93±6.03 | 24.90±1.68 | 0.667 |
| Glycine | 4.90±0.66 | 4.71±0.64 | 0.578 |
| myo_Insitol | 15.48±2.39 | 13.95±1.02 | 0.136 |
| Taurine | 5.06±1.44 | 4.07±0.84 | 0.130 |
| Choline | 0.19±0.07 | 0.28±0.09 | **0.046** |
| Asparate | 1.56±0.18 | 1.44±0.10 | 0.153 |
| Glutamine | 3.94±1.14 | 3.27±0.45 | 0.163 |
| Glutamate | 8.77±1.34 | 7.95±0.47 | 0.142 |
| GABA | 1.66±0.27 | 1.43±0.10 | **0.043** |
| NAA | 6.63±1.04 | 6.24±0.31 | 0.354 |
| Alanine | 0.58±0.16 | 0.67±0.16 | 0.278 |
| Lactate | 7.41±3.12 | 9.08±3.67 | 0.356 |

Data are shown as Mean±SD.

12. Cerebellum

|  | MIR(μmol/g) | Control(μmol/g) | *p* value |
| --- | --- | --- | --- |
| Cretine | 45.84±3.17 | 44.95±3.40 | 0.587 |
| Glycine | 2.37±0.44 | 2.28±0.29 | 0.607 |
| myo_Insitol | 17.27±2.60 | 16.83±2.17 | 0.705 |
| Taurine | 11.31±1.86 | 10.32±1.86 | 0.291 |
| Choline | 0.14±0.03 | 0.15±0.03 | 0.287 |
| Asparate | 1.54±0.19 | 1.54±0.24 | 0.943 |
| Glutamine | 7.84±0.94 | 7.53±0.68 | 0.451 |
| Glutamate | 14.96±1.86 | 14.82±1.20 | 0.857 |
| GABA | 1.90±0.26 | 1.90±0.23 | 0.973 |
| NAA | 7.91±1.21 | 7.87±0.80 | 0.930 |
| Alanine | 0.94±0.31 | 1.04±0.17 | 0.413 |
| Lactate | 9.51±3.33 | 10.97±4.66 | 0.469 |

Data are shown as Mean±SD.
